# Diagnosis and investigation of infertility causes in two female giant pandas using multimodal techniques: a case report

**DOI:** 10.3389/fvets.2026.1754538

**Published:** 2026-04-20

**Authors:** Ying Zhou, Honglin Wu, Jin Shu, Yingmin Zhou, Xiangqian Meng, Guiquan Zhang, Xinyu Lv, Guo Li, Ping Lei, Ming He, Zhen Lei, Qian Wang, Lingli Yi, Liu Yang, Penghao Li, Bo Luo

**Affiliations:** 1 China Conservation and Research Center for the Giant Panda, Key Laboratory of State Forestry and Grassland Administration on Conservation Biology of Rare Animals in the Giant Panda National Park, Chengdu, China; 2 Sichuan Jinxin Xi'nan Women's and Children's Hospital, Chengdu, China

**Keywords:** B-ultrasound, colposcopy, giant panda, hormone, hysteroscopy, infertility, laparoscopy

## Abstract

**Background:**

The advancement of artificial insemination techniques for giant pandas has significantly enhanced their breeding success. However, some pandas continue to experience difficulties in conceiving naturally, necessitating a thorough investigation into the underlying causes of infertility. This study aimed to perform pre-pregnancy examinations and diagnose infertility causes on giant pandas with infertility.

**Methods:**

A range of diagnostic methods, including behavioral observation, hormone assays, ultrasound imaging, laparoscopy, hysteroscopy, and colposcopy, were employed to conduct pre-pregnancy assessments and diagnose infertility in two giant pandas, designated as #A and #B.

**Results:**

Both pandas exhibited normal estrus behaviors, and their progesterone (P4) and estrone (E1G) levels during estrus paralleled those typically seen in successfully breeding individuals during estrus, remaining within the expected physiological range. Ultrasound evaluation of panda #A revealed a fluid accumulation surrounding the ovaries, with echogenicity noted along the walls of the adjacent fallopian tubes, suggesting the presence of hydrosalpinx, which was subsequently confirmed via laparoscopy. Hysteroscopic examination indicated that the vaginal and uterine structures of both pandas were normal.

**Conclusion:**

The estrous behaviors and hormonal profiles of the two infertile giant pandas were observed to be within normal ranges. The infertility in one of the subjects was attributed to the presence of hydrosalpinx.

## 1 Introduction

The giant panda (*Ailuropoda melanoleuca*), an endemic species to China, has garnered considerable attention as a flagship species for global biodiversity conservation. Revered as a national treasure, the giant panda is esteemed for its distinctive appearance and the limited number of individuals remaining in the wild. According to the Fourth National Survey Report on the Giant Panda (https://www.giantpandaglobal.com/giant-panda-news/the-results-of-the-fourth-giant-panda-survey/), there are 1,864 wild pandas

distributed across 33 local populations in China. The giant panda is classified as Vulnerable on the International Union for Conservation of Nature (IUCN) Red List, primarily due to its unique biological and physiological traits that heighten its risk of extinction (1, 2). In response, China has undertaken numerous conservation initiatives, including the establishment of giant panda reserves and the restoration of their natural giant panda habitats (3–5). Moreover, artificial insemination technology, initially developed to facilitate reproduction and enable selective breeding (6), has emerged as the predominant method in captive breeding programs for giant pandas globally, resulting in a significant increase in their population (7). Nonetheless, certain giant pandas, particularly those with high genetic merit, experience difficulties in conceiving naturally, leading to a notable reduction in genetic diversity. Consequently, it is imperative to diagnose and elucidate the causes of infertility in these pandas.

The reproductive cycle of giant pandas exhibits several unique and complex physiological characteristics. Female giant pandas are seasonal monoestrous animals, typically entering estrus between February and May, with parturition occurring from July to September (8, 9). The estrus period generally lasts 1 to 2 weeks, while the mating window is notably brief, spanning only 1 to 3 days, during which ovulation takes place (8, 9). Following ovulation, all female giant pandas experience an extended luteal phase, irrespective of pregnancy status (8, 9). Parturition occurs between 85 and 181 days post-mating, indicating a prolonged and highly variable gestation period (10). Furthermore, the absence of reliable external indicators of pregnancy presents significant challenges for pregnancy diagnosis and delivery prediction in both captive and wild populations of giant pandas.

Research on the causes of infertility in pandas remains limited. Behavioral observation and hormone testing are the two most prevalent methods used in the examination of infertile pandas (11, 12). Female pandas may undergo a comprehensive set of examinations, including medical history review, gynecological and ultrasound examinations, hormone assays, and transvaginal endoscopy; these procedures are collectively referred to as one-day diagnostics, or a one-stop fertility clinic (12). Non-invasive diagnostic techniques, such as B-ultrasound detection, laparoscopy, hysteroscopy, and colposcopy, have been utilized in females and are recommended for infertility evaluation. Ultrasound serves as a diagnostic tool to assess the morphology, position, and dimensions (length, width, and thickness) of the uterus, facilitating the evaluation of uterine dysplasia. Additionally, it enables the examination of the fallopian tubes, uterus, and ovaries for the presence of tumors, malformations, and endometrial polyps, thereby aiding in the diagnosis infertility (13). Laparoscopy provides a comprehensive assessment of uterine, tubal, and ovarian function and is considered the gold standard for diagnosing tubal and peritoneal abnormalities that may compromise fertility (14, 15). Hysteroscopy, a minimally invasive procedure, permits direct visualization of the endocervical canal, uterine cavity, endometrium, and tubal ostia (16). Colposcopy, employing a gynecological endoscope, is primarily used for the auxiliary diagnosis and evaluation of vulvar, vaginal, and cervical intraepithelial lesions, as well as early cervical cancer and other early lesions of the lower genital tract. This procedure allows for the direct observation of the vascular morphology and epithelial structure in the exposed vagina and cervix, facilitating the detection of abnormal epithelial and vascular patterns associated with malignancy.

In the present study, to perform pre-pregnancy examinations and diagnose infertility causes on giant pandas, a comprehensive array of techniques, including behavioral observation, hormone analysis, ultrasound imaging, laparoscopy, hysteroscopy, and colposcopy, were used in giant pandas. The results showed that the estrous behaviors and hormonal profiles of the two infertile giant pandas were observed to be within normal ranges. Ultrasound evaluation of panda #A revealed a fluid accumulation surrounding the ovaries, with echogenicity noted along the walls of the adjacent fallopian tubes, suggesting the presence of hydrosalpinx, which was subsequently confirmed via laparoscopy. Hysteroscopic examination indicated that the vaginal and uterine structures of both pandas were normal. The findings from this research are anticipated to provide valuable insights for the conservation and effective protection of giant pandas and other endangered species, thereby contributing to the preservation of biodiversity.

## 2 Methods

### 2.1 Animals

This study involved two adult female giant pandas, identified by pedigree numbers #A and #B. The study period was 2000 to 2022. At the time of the study, Panda #A was 22 years old, born in 2000, in good health, and had no significant medical history. She reached physical maturity (being fully grown) at age 6. At age 9 (in 2009), she experienced her first natural estrus, mated with Wu Gang and gave birth to 1 offspring. She did not exhibit estrus from ages 10 to 13. At age 14 (in 2014), she mated with Lu Lu & Wu Gang in estrus and did not produce offspring. She did not exhibit estrus from 15 to 17 years of age. At age 18 (in 2018), she naturally mated with Wu Gang & Jin Ke and did not produce offspring; at age 19 (in 2019), she naturally mated with Bai Yang & Zi Yan & Jin Ke and did not produce offspring; at age 20 (in 2020), she naturally mated with Zi Yan and did not produce offspring. Between the ages of 21 and 22, she did not exhibit signs of estrus. Subject #B, who was 15 years old, born in 2007, was in poor bodily condition but had no history of major illnesses. Her physiological maturity (being fully grown) was reached at age 6. At age 7 (in 2014), she experienced her first natural estrus and underwent artificial insemination. At age 8 (in 2015), she was not mated in estrus. At age 9 (in 2016), she naturally mated with An An & Tai Shan; at age 10 (in 2017), she underwent artificial insemination with Tai Shan & Tong Tong; at age 11 (in 2018), she naturally mated with Bai Yang; at age 12 (in 2019), she naturally mated with Bai Yang & Ao Ao; at age 13 (in 2020), she naturally mated with Tai Shan & Ao Ao; at age 14 (in 2021), she was not bred; at age 14 (in 2021), she was in estrus naturally but was not bred. The above records were in normal estrus every year, and as of 2022, she (Subject #B) had never given birth.

All the above males had natural mating ability and had produced multiple offspring through natural mating (males mated with Subject #A and B had normal fertility). The reproductive and fertility history of the pandas was exhibited in Supplementary Table 1.

The basic medical information of the two pandas was provided in Supplementary Table 2. Subject #A (examined on 15 March 2022) showed no signs of estrus on that day and was in a physiological state without estrus. The laparoscopic time of subject #B was 34 h after the estrogen peak, which was in the late peak of estrus (still in the mating period).

### 2.2 Behavioral observation

The study employing the whole event recording method to observe and record the estrus behaviors of giant pandas through surveillance video. Based on the definitions and findings of previous studies (17–19), the behaviors of giant pandas, such as activity levels, splashing, vocalizations (birdsong and bleating), vulvar changes, tail lifting, and mating acceptance, were categorized and recorded on a scale from 1 to 3. The overall rating of estrus behavior was categorized into four levels: excellent, medium, poor, and none. Rating of excellent and medium were classified as normal, whereas ratings of poor and none were deemed abnormal. Subject #A showed normal estrus behavior, successfully completed natural mating in 2020, and did not show estrus behavior in 2022. Subject #B shown normal estrus behavior every year from 2016 to 2022.

### 2.3 Enzyme-linked immunosorbent assay

From the onset of estrus behavior in female subjects, urine samples were was collected 1 to 3 times daily until the conclusion of estrus. The occurrence of the following behaviors indicated the beginning of estrus: When the amount of individual activity increased, occasionally accompanied by rubbing Yin, splashing and other behaviors, and the vulva began to swell and open; In addition, estrogen broke through the basal value and gradually increased, with E1G/P4 ≥ 1. The end of estrus was generally defined as the female no longer displayed mating behavior after reaching the peak estrus period or after mating, which generally ended 48 h after the estrogen peak. The levels of hormones were measured immediately after urine collection. The urine samples were centrifuged at 2,500 × g for 10 min at 4 °C, and 500 μl of the supernatant was extracted for hormone analysis using enzyme-linked immunosorbent assay (ELISA). The concentrations of estrone (E1G) and progesterone (P4) were quantified using monoclonal antibodies specific to E1G (R522-2, Coralie Munro, UC Davis, USA) and P4 (CL425, UC Davis, USA).

### 2.4 Preoperative preparation

#### 2.4.1 Basic anesthesia

Prior to anesthesia, the giant pandas were fasted from food and water for 8 to 24 h. Atropine (1 mg) was administered 20 min before anesthesia, followed by an intramuscular injection of ketamine at a dosage of 8 to 10 mg/kg for basic anesthesia.

#### 2.4.2 Preanesthesia medication and induction of anesthesia

Upon arrival in the treatment room, midazolam was employed as a muscle relaxant. Tracheal intubation was performed concurrently with the establishment of venous access.

#### 2.4.3 Preparation of the skin

The abdomen fur was shaved from the perineum to the costal margin. Following the completion of skin preparation, the giant pandas were transferred to the operating room for laparoscopic and other examinations.

#### 2.4.4 Inhalation anesthesia

The isoflurane (R510-22, RWD, Shenzhen, China) volatile tank gas concentration was adjusted to 1.0%−1.5% based on the operational requirements, with an oxygen flow rate of 2 L·min^−1^. In the event of increased heart rate or blood pressure during skin incision or other physical responses, such as traction of internal organs, suturing, or other procedures, the isoflurane concentration could be increased appropriately, and the oxygen flow adjusted accordingly.

#### 2.4.5 Intraoperative hydration

The daily water requirements for the animal body were the amount of water loss plus maintenance fluid plus progressive loss. Water loss (mL) was determined by the formula: body weight (kg) × degree of dehydration (%) × 1,000. The maintenance volume was set at 40 to 60 mL·kg^−1^·d^−1^.

### 2.5 Ultrasonic examination

After the administration of general anesthesia and skin preparation, the giant panda was positioned supine, with limbs relaxed, feet slightly externally rotated, and the skin beneath the bilateral nipples fully exposed, ensuring that the entire abdominal musculature was relatively relaxed. Initially, a routine pelvic ultrasound examination was conducted. Specifically, the longitudinal section of the probe provided visualization of the uterus's position and shape, as well as the endometrial thickness and type. Subsequently, the probe was rotated to examine both ovaries in longitudinal and transverse sections, documenting the size and number of follicles on each side. The procedure utilized a Samsung HS50 ultrasound diagnostic instrument, equipped with a convex array probe operating at 1~7 MHz, with a mechanical index of 0.38–0.42. The gain and depth settings were maintained consistently, and no contrast agent was used.

### 2.6 Laparoscopic and hysteroscopic examination

The giant panda was placed in a supine positioned supine and prepared for aseptic abdominal surgery. A 10 mm incision was made 1.0–1.5 cm above the panda's first nipple (Right and caudal thoracic nipple). Following the incision of the peritoneal layer, a trocar was inserted, and 3.5–4 L of CO_2_ was introduced while maintaining an intraperitoneal pressure of 10–12 mmHg using a pneumoperitoneum machine (26430508–1, STORZ, Tuttlingen, Germany) to achieve pneumoperitoneum. A laparoscope (VQ812558 P\VQ811957 P, 5029319, STORZ) was introduced to facilitate observation of the pelvic region of the abdomen. Subsequently, two incisions, each measuring 5 mm were made to the left of the ventral midline of the giant panda, spaced 10 mm apart. Utilizing a laparoscopic monitoring system (E240A, STORZ), two 5 mm punctures were created in the abdominal cavity, allowing for the insertion of 5 mm non-invasive grasping forceps, suction devices, and bipolar electrocoagulation forceps. The pelvic genitalia of the giant panda were examined via the laparoscope. Upon completion of the surgical procedure, the abdominal cavity was re-examined with the laparoscope to confirm the absence of bleeding, after which the laparoscope was withdrawn. The vent valve of the surgical cannula was opened to release carbon dioxide gas. The puncture device was removed, and the peritoneal layer, fascial layer of the abdominal wall, and subcutaneous and skin layers were sutured discontinuously using 2–00 absorbable sutures. After the examination, the giant pandas received an oral antibiotic regimen of 6 capsules of amoxicillin (0.5 g/capsule) administered twice daily for a duration of 5 days. In addition, the giant pandas underwent hysteroscopy prior to the laparoscopic examination.

### 2.7 Statistical analysis

The data (the E1G and P4 levels) were analyzed using SPSS software version 20.0 (IBM, Armonk, New York, USA) and were presented as the means ± standard deviations (SD). A Student's *t*-test was employed to assess significant differences, with a significance threshold set at *p* < 0.05.

## 3 Results

### 3.1 Results of behavioral observation

The estrus behaviors of both giant pandas were observed to be normal, and the overall rating of estrus behavior was detailed in Table 1.

**Table 1:** The overall rating of estrus behavior of the two giant pandas (Estrus behavior grade 1–3, accuracy level 0.5).

Individual	Year of estrus	Amount of activity	Splashing	Rubbing	Birdsong	Bleating	Vulvar changes	Tail lifting	Acceptance of mating	General assessment of estrus behavior
#A	2009	2	1.5	1.5	2	2.5	2.5	2.5	3	Excellent
	2014	2.5	1.5	1.5	1.5	2.5	2.5	2.5	3	Excellent
	2018	3	2	2	1.5	2	3	2.5	3	Excellent
	2019	3	1.5	2	1.5	2.5	2	2.5	3	Excellent
	2020	2.5	1.5	1.5	1.5	2.5	2	2.5	3	Excellent
#B	2015	1.5	0.5	1	1	1.5	1.5	1.5	1.5	Medium
	2016	2	1	1	1.5	1.5	2	1.5	2	Medium
	2017	2	1	1	1.5	2	2.5	2	3	Excellent
	2018	2.5	1	1.5	1.5	2	2.5	2.5	3	Excellent
	2019	2.5	1	1.5	1.5	2.5	2.5	2.5	3	Excellent
	2020	2	1	1.5	1.5	2.5	2.5	2.5	3	Excellent
	2021	2.5	1.5	1.5	2	2.5	2.5	2.5	3	Excellent
	2022	2	1	1.5	1.5	2.5	2.5	2.5	3	Excellent

### 3.2 Examination of the E1G and P4 levels

**Figure 1:**
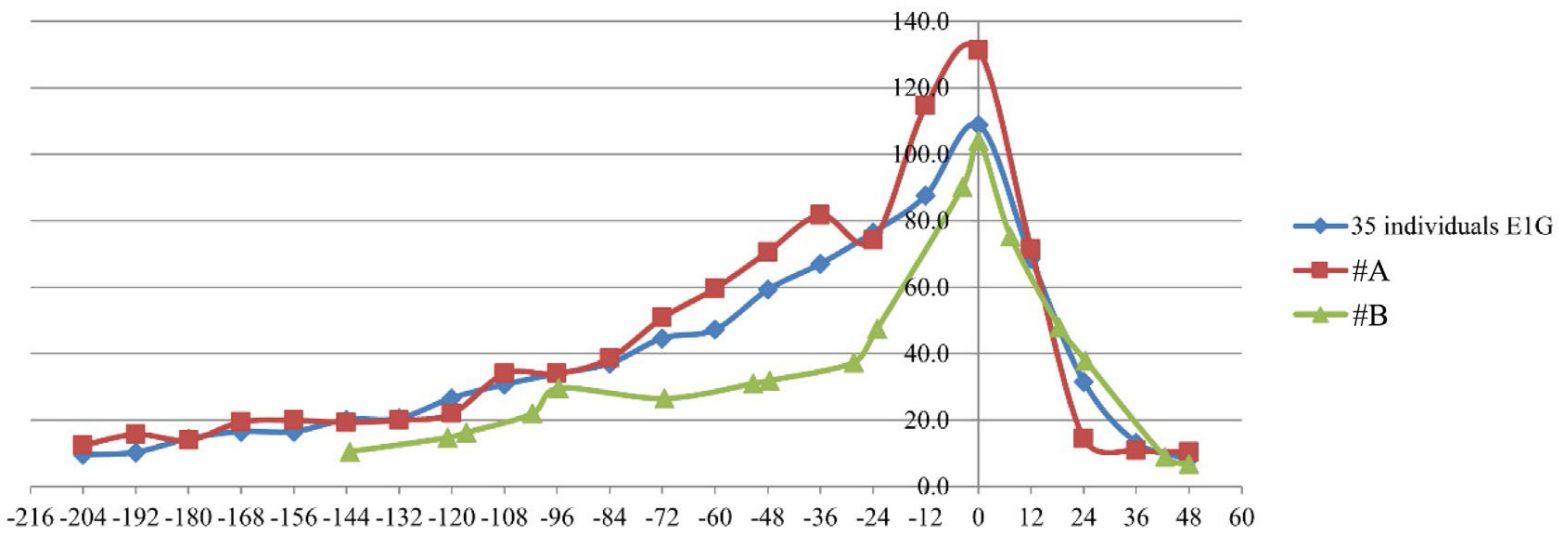
The E1G levels in pandas #A and #B in comparison to those in 35 individuals with a history of pregnancy. The units of measurement were ng/mg·Cr.

**Figure 2:**
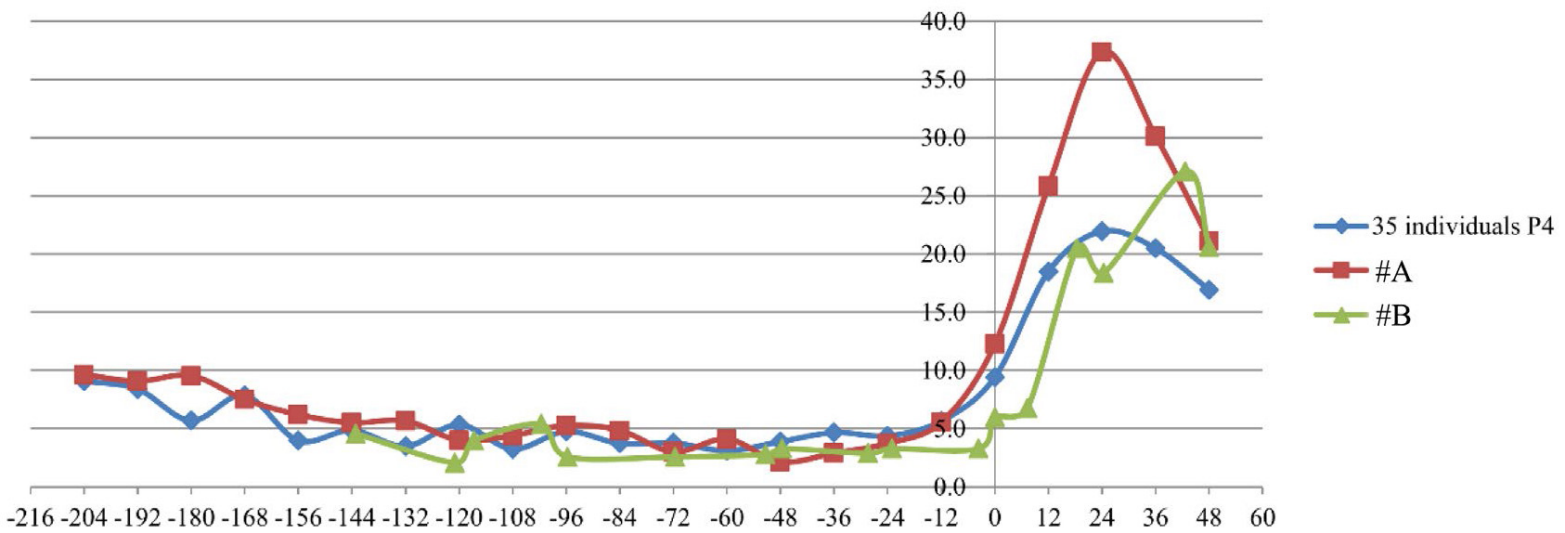
P4 levels in #A and #B compared with those in 35 individuals with a history of pregnancy. The units of measurement were ng/mg·Cr.

Estrus hormone data for panda #A from 2019 to 2022 and for panda #B from 2020 were analyzed and compared with data from 35 individuals with a history of pregnancy during their estrus. The trends of E1G and P4 levels during estrus were analogous to those observed in normal pregnant individuals, and the hormone levels remained within the normal range (Figures 1, 2).

### 3.3 Results of the ultrasound examination

**Figure 3:**
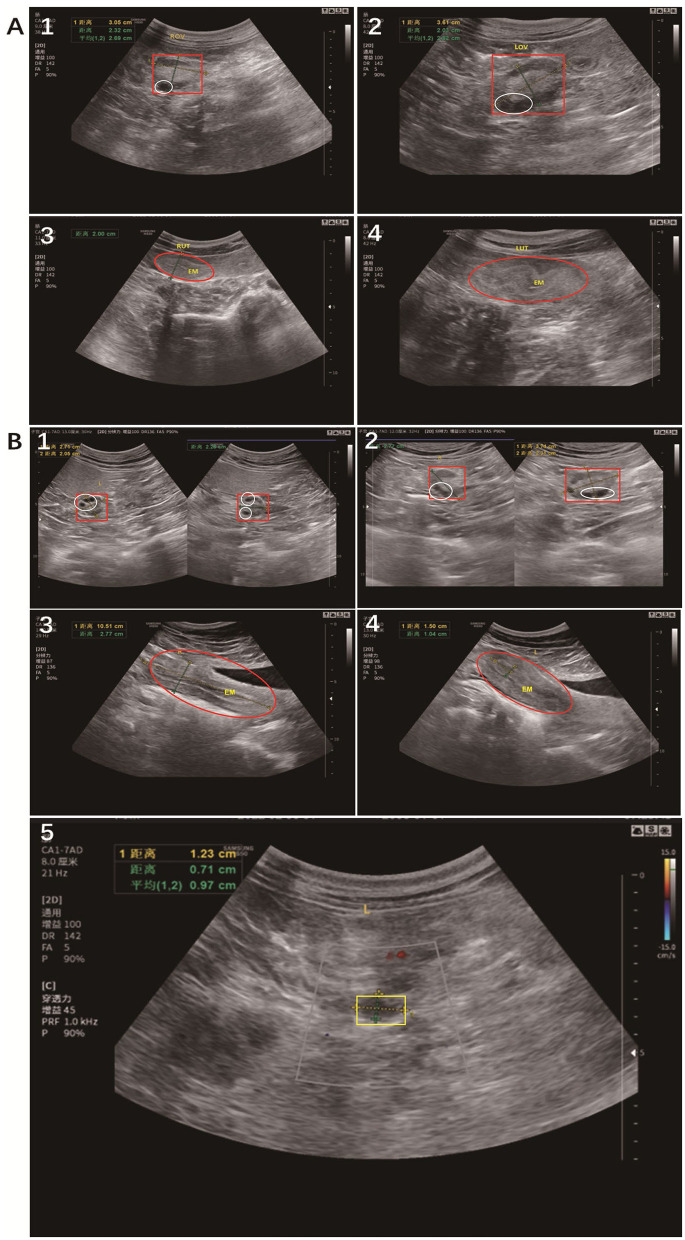
Ultrasound imaging of the giant panda uterus during ovulation. A, Panda #A, images **(A1)** and **(A2)** depicted the right and left ovaries, respectively; **(A3)** and **(A4)** illustrated the right and left uterus and endometrium, respectively. B, Panda #B, images **(B1)** and **(B2)** showed the right and left ovaries, respectively; **(B3)** and **(B4)** displayed the right and left uterus and endometrium, respectively; **(B5)** indicated a suspicion of hydrosalpinx. Red rectangle indicated the ovaries, and the white oval indicated the follicles; Red oval indicated the uterus, and the white lines were endometrium (Designated as EM); Yellow rectangle indicated the location of hydrosalpinx. ROV, Right ovarian volume; LOV, Left ovarian volume; RUT, Right uterine transversa; LUT, Left uterine transversa; EM, Endometrium; R, Right; L, Left.

Ultrasound examinations indicated that the uteri of both giant pandas exhibited a bicornuate shape, characterized by two distinct uterine cavities. The uterine cavities were relatively slender, each possessing an independent endometrium and a depression in the serous layer at the fundus. No clear demarcations were observed between the cervix and the uterine body. As the endometrial structure underwent changes, corresponding alterations in echogenicity were noted. During the ovulatory phase, peristaltic waves were observed within the endometrium (Figures 3A1–4, B1–4). In the course of the pelvic ultrasound examination, Panda #B exhibited a fluid-filled region surrounding the ovaries, with echogenic signals detected from the walls of the fallopian tubes adjacent to this fluid accumulation, suggesting the presence of hydrosalpinx (Figure 3B5). In contrast, no sonographic visualization of the fallopian tubes was achieved for the other giant panda throughout the entire ultrasound assessment.

### 3.4 Results of laparoscopic examination

**Figure 4:**
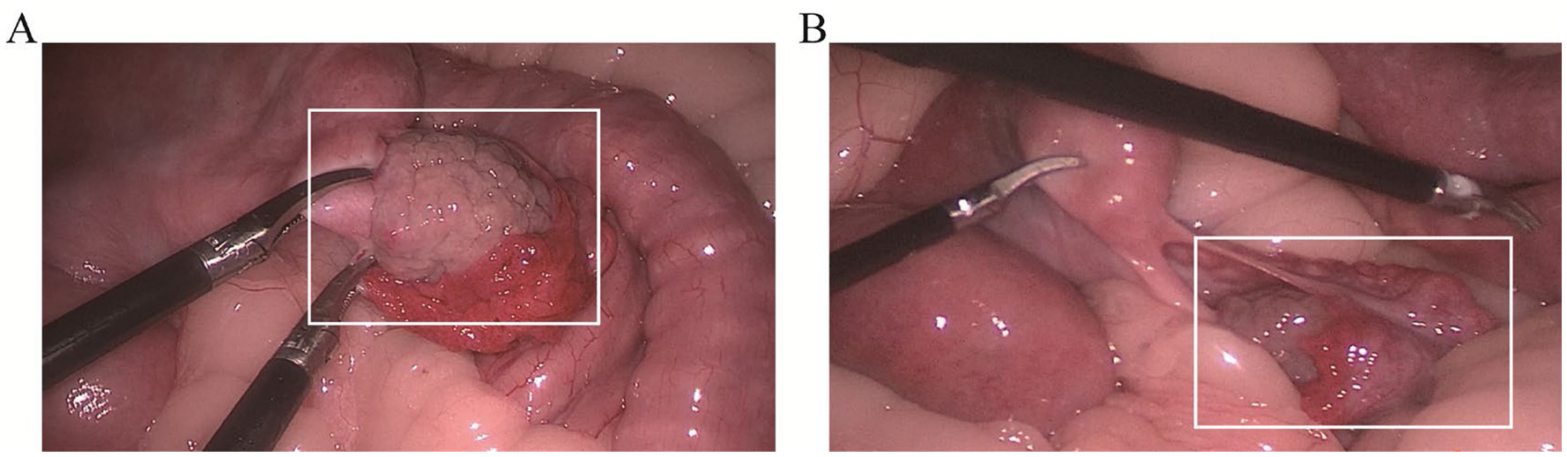
Laparoscopic examination of the two giant pandas. Panda #A **(A)** and Panda #B **(B)** exhibited the ovaries of two giant pandas, with an estimated size about 3 × 3 × 3 cm. The ovaries were ruddy color, good blood supply, and furrow and primitive follicle like processes in the ovarian cortex. Oviduct attachment was seen beside the ovary, and the fimbriae of the fallopian tubes were rich in mucosa without adhesion around them. White rectangle indicated the ovaries.

Regarding uterine morphology, the uterine length on both sides measured approximately 18–20 cm, with a width of 4–5 cm, and the uterine surface appeared pink with pronounced folds. The ovarian dimensions for the pandas were approximately 3 × 3 × 3 cm. Several 2–5 mm follicles were identified on the surface of panda #A (Figure 4A); however, panda #B exhibited no have follicles, with only a few miliary vesicles present on the ovarian surface (Figure 4B). The fallopian tubes of the giant pandas measured approximately 5–7 mm in length, with some appearing nodular and others adhering to the ovaries and adjacent tissues. Laparoscopic examination revealed no significant abnormalities in the fimbriae of the fallopian of either giant panda.

### 3.5 Results of hysteroscopic examination

**Figure 5:**
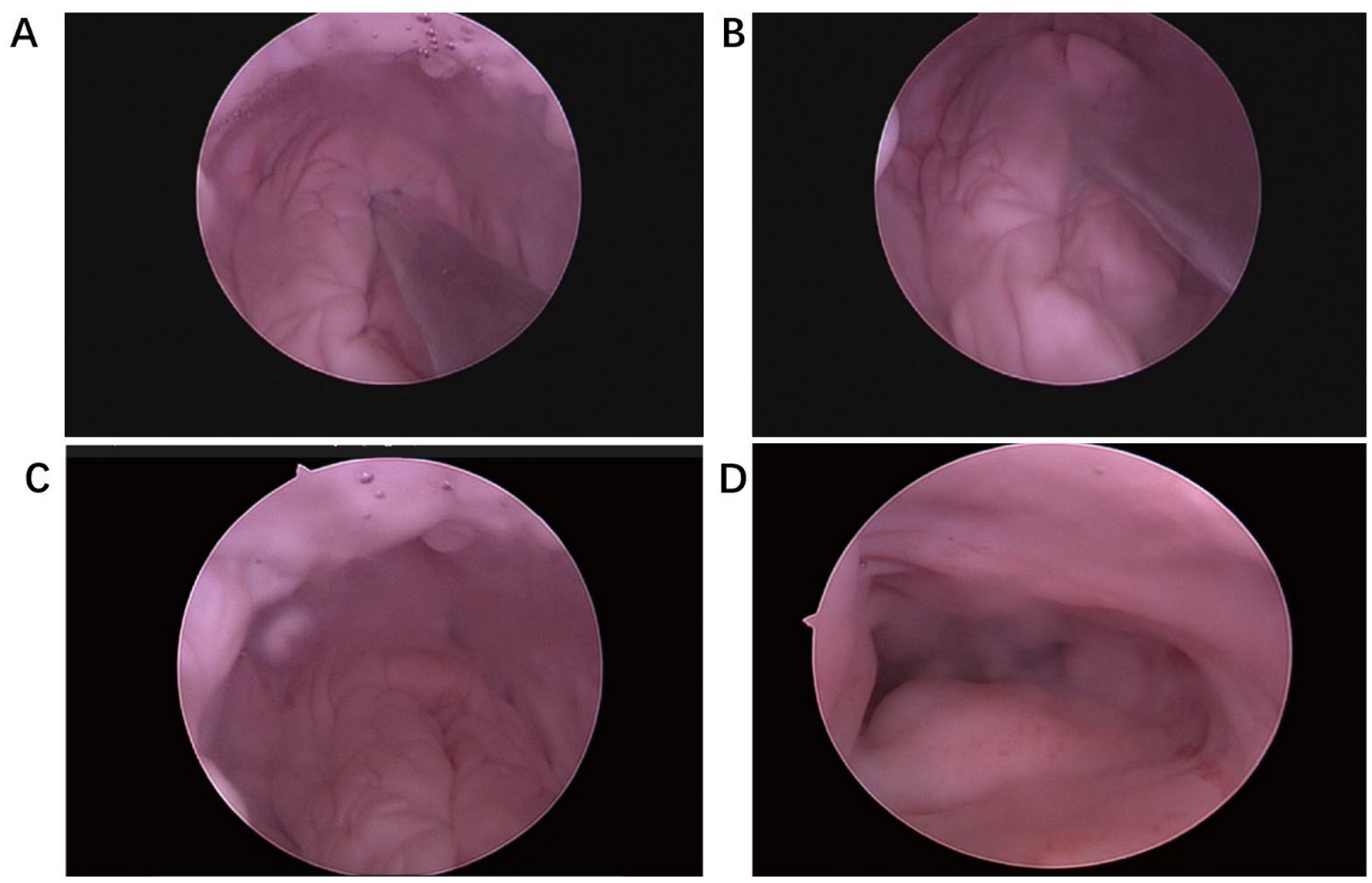
Hysteroscopic examination of the two giant pandas. **(A, B)** The hysteroscopy of the probe into the cervical orifice. **(C)** The morphology of the cervix under hysteroscopy: a hill-like oval bulge at the top of the vagina, 20 (long) *15 (wide) *8 (high) mm, with irregular gully on the cervical surface and no obvious cervical entrance. **(D)** The hysteroscope entering the cervical canal, and the four walls of the cervical canal were wrinkled and ruddy.

The vaginal cavity of the giant pandas was approximately 5 cm in depth and 3–4 cm in width. The urethral and vaginal openings were observable at the apex of the genital cavity, and the space between the 1 cm × 5 mm hysteroscope and the panda's vagina was discernible. The vaginal wall appeared pink with folds, and the surrounding area lacked distinct cervical tissue. The posterior wall exhibited convex folds 2 cm below the vaginal apex, with no apparent perforations on the surface (Figure 5A–C). Under ultrasound guidance, probes used for artificial insemination were repeatedly applied at the center of the convex fold. The probe gently entered the left uterine cavity (Figure 5A, B). The uterine cavity was barrel-shaped, with a pink inner lining (Figure 5D). Hysteroscopy examination of the uteruses of two pandas revealed no apparent abnormalities.

## 4 Discussion

As China's most iconic species, the giant panda garners significant attention due to its limited population and low natural reproductive rate. Although advancements in artificial insemination have significantly enhanced breeding efforts, some individuals still face challenges in conceiving naturally. Therefore, diagnosing and understanding the causes of infertility in pandas is imperative. A comprehensive diagnostic approach, integrating various modalities, is crucial for a thorough understanding and effective management of female infertility. The diagnostic process should systematically commence with a detailed medical history and physical examination which will inform subsequent hormonal and imaging assessments.

First, infertility should be confirmed by inquiring about the number of attempts at breeding the patient has submitted to. Patient age is an important prognostic factor (20). It is important to obtain information about the age of puberty, sexual history, previous pregnancies, duration of infertility, and previous treatments. In the present study, in 17 years of adult life, panda #A had three natural estrus cycles, and two induced estrus cycles. Each estrus behavior was typical, but achieving only two births after five attempts at breeding, indicated low fertility. Panda #B had a total of eight natural estrus cycles in the ten years after adulthood. The pandas exhibited normal estrus behavior during each estrus cycle and despite seven individual breeding attempts had not conceived, suggesting infertility of undetermined origin. Giant panda reproduction is predominantly seasonal, with females typically entering estrus only once a annually during the spring. In captivity, female giant pandas generally reach sexual maturity by the age of five, although some individuals may begin to experience estrus as early as ages three to four. Panda #A has consistently maintained good health without any major illnesses; however, she did not enter estrus until the age of nine. This delay may be attributed to late sexual maturity or other factors inhibiting her estrous cycle. Following her initial successful breeding, Panda #A has not exhibited estrus for several years, and despite undergoing estrus and mating, she has not achieved successful reproduction. This suggests the presence of a factor impending Panda #A's estrous cycle and delaying her initial estrus until the age of nine. Conversely, Panda #B first entered estrus at the age of seven and subsequently experienced annual estrus cycles accompanied by pseudopregnancies. It has been established that giant pandas can experience pseudopregnancies even in the absence of mating post-ovulation. This indicates that panda #B is likely to ovulate every year, and the probability of problems related to estrus and subsequent ovuluation is unlikely but may be related to fertilization, implantation and maintenance of pregnancy thereafter.

Estrone is one of the three primary endogenous estrogens. Post-pregnancy, progesterone facilitates the implantation of the fertilized egg into the uterus and supports the progression of pregnancy, contingent upon estrogenic activity. The analysis of E1G and P4 levels indicated that the E1G level trends during estrus were analogous to those observed in normally pregnant animals, with hormone levels remaining within the standard range. This suggests that hormonal factors related to infertility are normal in the female giant panda.

In the present study, findings revealed that panda #A exhibited a fluid-filled area surround the ovaries, accompanied by an echo from the fallopian tube walls adjacent to this fluid, raising suspicion of hydrosalpinx. Laparoscopy, a procedure characterized by its safety and minimal complication risk, allows for the visual apprailsal of uterine, tubal, and ovarian structures, often circumventing the need for unnecessary laparotomy. The application of laparoscopic surgery in wildlife medicine is increasingly popular. Specifically, in reproductive surgery, laparoscopic oophorectomy, ovarian hysterectomy, and salpingectomy have been used for treatment or sterilization across various wildlife species. Laparoscopic surgery in wild animals offers advantages such as smaller incisions and reduced recovery times compared to laparoscopic procedures in humans. This technique is particularly effective for assessing reproductive status, including ovarian anatomy and function, performing direct visual biopsies of internal organs, determining sex in certain avian species, and serving as a surgical method for fertility control (21, 22). This study provides a comprehensive overview of the application of laparoscopic surgery in Chinese giant pandas, contributing further evidence to support the feasibility and safety of laparoscopic reproductive examinations in the field of giant panda infertility investigations.

Hysteroscopy, with its capacity to magnify the endometrial cavity, is instrumental in detecting minute changes. It is recommended as a first-line technique for evaluating the uterine cavity in infertile women undergoing assisted reproductive procedures (23). In this study, hysteroscopy was performed on two giant pandas, revealing normal vaginal and uterine cavity conditions. Due to the limited length of the human hysteroscope, it penetrated the uterine cavity of the giant panda to a depth of only approximately 5 cm, insufficient to reach the uterine fundus. Consequently, the efficacy of uterine distension was suboptimal, preventing complete distension of the uterine cavity.

Notably, employing multiple diagnostic methods may be optimal. For instance, the combination of laparoscopic examinations with transvaginal B-ultrasound scans has demonstrated utility in the etiological investigation of female infertility (24). The concurrent use of hysteroscopy and laparoscopy has been reported as both safe and effective for infertility diagnosis, as noted by Dawle et al. (25) in 2014 and Mehta et al. (26) in 2016. However, Shobha et al. (27) later suggested in the same year that there is room for improvement in operational skills. Hysterolaparoscopy is recognized as the gold standard for diagnosing infertility in women (28). Additionally, imaging examinations and blood tests are frequently used as complementary methods in the assessment of female infertility. Imaging examinations provide structural insights into the uterus, ovaries, and fallopian tubes, while blood tests yield hormonal information. Together, these diagnostic tools offer a comprehensive evaluation of reproductive health.

## 5 Conclusion

This study systematically investigated the causes of infertility in two female giant pandas through a multimodal diagnostic approach that integrated behavioral observation, hormone assays, ultrasound, laparoscopy, and hysteroscopy. Both individuals exhibited normal estrus behaviors and hormonal profiles, with P4 and E1G levels comparable to those of fertile pandas. Structural abnormalities were identified in panda #A, where ultrasound and laparoscopic examinations confirmed the presence of hydrosalpinx (fluid-filled, dilated fallopian tubes), a condition likely impeding gamete transport and embryo implantation. Conversely, no anatomical anomalies were detected in panda #B, despite recurrent false pregnancies, suggesting potential functional or molecular defects in ovulation, fertilization, or endometrial receptivity. The integration of advanced diagnostic tools, particularly laparoscopy and hysteroscopy, has been pivotal in identifying complex reproductive disorders. However, technical limitations have emerged due to anatomical differences between humans and pandas. These findings emphasize hydrosalpinx as a treatable cause of infertility through surgical intervention or assisted reproductive technologies, while highlighting the necessity for further investigation into unexplained infertility cases, such as those related to oocyte quality or immunological factors in #B. One of the obvious deficiencies of this study were the small sample size, including only 2 individuals, as well as only some or only 1 cycle followed. Despite the small sample size and the challenges associated with adapting human-derived instruments, this multidisciplinary framework offers a strategic approach for enhancing captive breeding programs, addressing infertility in genetically valuable individuals, and preserving the genetic diversity of endangered species, such as the giant panda. Moreover, although the information on male pandas mated to pandas A and B was exhibited in this study, the quality of the semen and the many possible causes of non-conception related to male factors were not elaborated in detail. This was one of deficiency of this study. Furthermore, since there is no corresponding standard and reference for infertility in pandas, and the preliminary judgment is only based on the actual situation, such as no pregnancy in x years, this study cannot comprehensively speculate on the possibility of infertility in pandas. Future studies should aim to expand diagnostic protocols to incorporate molecular analyses and develop species-specific adaptations of reproductive technologies.

## Data Availability

The original contributions presented in the study are included in the article/Supplementary material, further inquiries can be directed to the corresponding authors.
