# Supplementary material for: Diagnosis and investigation of infertility causes in two female giant pandas using multimodal techniques: a case report
Source: Front Vet Sci. 2026 Apr 20;13:1754538. doi: 10.3389/fvets.2026.1754538 (PMC13137947; doi:10.3389/fvets.2026.1754538)
Supplement: Supplementary file 1 [file Table_1.docx]

Supplementary Table 1. The reproductive and fertility history of the pandas

| Subjects | Year | The reproductive history | The fertility history |
| --- | --- | --- | --- |
| Panda #A (born in 2000) | 2009 | Yes | Yes |
|  | 2014 | Yes | No |
|  | 2018 | Yes | No |
|  | 2019 | Yes | No |
|  | 2020 | Yes | No |
|  | 2021 | No | / |
|  | 2022 | No | / |
| Panda #B (born in 2007) | 2014 | Yes | No |
|  | 2015 | Yes | No |
|  | 2016 | Yes | No |
|  | 2017 | Yes | No |
|  | 2018 | Yes | No |
|  | 2019 | Yes | No |
|  | 2020 | Yes | No |
|  | 2021 | Yes | No |
|  | 2022 | Yes | No |
