# Supplementary material for: Diagnosis and investigation of infertility causes in two female giant pandas using multimodal techniques: a case report
Source: Front Vet Sci. 2026 Apr 20;13:1754538. doi: 10.3389/fvets.2026.1754538 (PMC13137947; doi:10.3389/fvets.2026.1754538)
Supplement: Supplementary file 2 [file Table_2.docx]

Supplementary Table 2. Health Check and Evaluation Form of panda #A

Date: 15 March 2022

|  | Age/Years | Weight/kg | Stool weight /kg | Mental status classification | Blood routine index detection (18 items) | Blood biochemical indexes (14 items) | Detection of trace elements in blood (5 items) | Thyroid function test (6 items) | Routine urine test (18 items) |
| --- | --- | --- | --- | --- | --- | --- | --- | --- | --- |
| Value of monitoring | 21 | 123.8 | 9.3 | 4 |  |  |  |  |  |
| Range | 0~38 | 78~138 | 0~20 | 1~5 |  |  |  |  |  |
| Evaluation | Age of childbearing | Being overweight | Normal | Normal | Normal | Normal | Normal | Normal | Normal |
| Overall evaluation | Healthy, overweight | | | | | | | | |

Health Check and Evaluation Form of panda #B

Date: 6 February 2022

|  | Age/Years | Weight/kg | Stool weight /kg | Mental status classification | Blood routine index detection (18 items) | Blood biochemical indexes (14 items) | Detection of trace elements in blood (5 items) | Thyroid function test (6 items) | Routine urine test (18 items) |
| --- | --- | --- | --- | --- | --- | --- | --- | --- | --- |
| Value of monitoring | 14 | 91.5 | 4.5 | 4 |  |  |  |  |  |
| Range | 0~38 | 78~138 | 0~20 | 1~5 |  |  |  |  |  |
| Evaluation | Age of childbearing | Being underweight | Less (in heat) | Normal | Normal | Normal | Normal | Normal | Normal |
| Overall evaluation | Healthy, underweight | | | | | | | | |
